# Attitude of tunisian psychiatry residents toward internet searches for patient informations

**DOI:** 10.1192/j.eurpsy.2024.1198

**Published:** 2024-08-27

**Authors:** Y. Ben Youssef, M. Lagha, S. Boudriga, M. Methni, I. Ben Romdhane, W. Homri, R. Labbane

**Affiliations:** Department C, El Razi Hospital, Mannouba, Tunisia

## Abstract

**Introduction:**

In the age of digital information, the volume of personal information available online continues to grow. Examining patients’ online profiles has become common for various reasons especially in psychiatry, despite ethical concerns. Therefore, it is interesting to explore the attitudes of tunisian psychiatric residents in this regard.

**Objectives:**

This study aimed to identify the purposes that make psychiatry residents consult their patients’ profiles on social media and to evaluate the consequences of being friends with them or following them on the treatment course and on the doctor-patient relationship.

**Methods:**

This was a cross-sectional descriptive study from August to September. A questionnaire on Google form was distributed to psychiatry Tunisian residents.The study evaluated the frequency and causes of patient profiles consultation on social networks, its role and impact in the doctor-patient relationship

**Results:**

The study population included 53 psychiatry residents with a mean age of 28 (+-5) years and a sex ratio of 0.127. Among the responders, 53 % were in their first or second year of residency. And the predominant workplace was El Razi Hospital : a university hospital.

For the frenquency of patient profiles consultation on social networks : 87% of treating psychiatrists declared consulting their patients’ profiles on social media at least once. The purposes of consulting patient’s profiles noted in our study were: looking for signs of pre-morbid functioning (n=32), looking for clinical features of the current episode (n=30). They do it also to verify the informations provided by the patient (n= 18) ,have an idea of their private lives (marital status ,employment, hobbies,..)(n=11) ,or locate a family member (n=5). It can be also out of curiosity (n=21). And this made the psychiatry residents empathetic towards the patient (n=10) .

But, in 91% of cases, patient’s permission was not taken .

Moreover, 4 of treating psychiatrists declared being friends with their patients or following their profiles on social media. Two of them regret it. The friend or follow request was an initiative from the patient, in all cases.

**Conclusions:**

The attitudes of psychiatry residents regarding the consultation of patients’ profiles on social networks were not clear. However, as the boundaries of the digital doctor-patient relationship remain undefined, it is imperative to develop clear guidelines and educational resources.

**Disclosure of Interest:**

None Declared

